# Secondary HIV Cholangiopathy as a Cause of Acute Cholangitis: A Case Report

**DOI:** 10.7759/cureus.90991

**Published:** 2025-08-25

**Authors:** Guillermo Avalos Gonzalez, Mariana Carolina Gasga Velasco, Paola V Rosales Verduzco, Ana Laura Rodríguez Hernández, Pablo Yael Enríquez Plascencia, Dina Paola Gutierrez Plascencia, Gerardo Garcia Santiago

**Affiliations:** 1 Internal Medicine, Hospital Regional Dr. Valentín Gómez Farías - Institute for Social Security and Services for State Workers or Civil Service Social Security and Services Institute (ISSSTE), Zapopan, MEX; 2 General Medicine, Universidad Autónoma de Guadalajara, Guadalajara, MEX

**Keywords:** antiretroviral therapy, benign biliary disease, biliary stricture, cholestasis, endoscopic retrograde cholangiopancreatography(ercp), hiv/aids complications, hiv cholangiopathy, opportunistic infections

## Abstract

HIV-associated cholangiopathy (HAC) is a rare but clinically important hepatobiliary complication occurring in patients with advanced immunosuppression, particularly those with cluster of differentiation 4+ (CD4+) counts below 100 cells/μL. It is most often triggered by opportunistic infections and can present as a spectrum of biliary abnormalities that result in cholestasis and obstruction. We present the case of a 58-year-old man with a history of HIV infection (B24) under regular follow-up, with an undetectable CD4 count and elevated viral load, who presented to the emergency department with jaundice, severe right upper quadrant and epigastric abdominal pain, nausea, vomiting, and a 10 kg weight loss over the past month. During hospitalization, a complex distal common bile duct (CBD) stricture was identified, accompanied by acute cholangitis, obstructive pancreatitis, and acute kidney injury (AKI) on a background of chronic kidney disease (CKD). The patient underwent endoscopic retrograde cholangiopancreatography (ERCP) with biliary stent placement, resulting in clinical improvement. HIV-associated cholangiopathy was confirmed as the underlying diagnosis. This case highlights the need to consider HAC in immunosuppressed patients presenting with cholestatic liver profiles and supports the importance of timely endoscopic intervention and multidisciplinary management.

## Introduction

HIV-associated cholangiopathy (HAC) is a rare but clinically significant hepatobiliary complication that primarily affects patients with advanced immunosuppression, particularly those with cluster of differentiation 4+ (CD4+) T-cell counts below 100 cells/µL [1]. First described in the 1980s before the widespread use of antiretroviral therapy (ART), HAC encompasses a spectrum of biliary abnormalities, including sclerosing cholangitis, papillary stenosis, and multifocal strictures of the intrahepatic and extrahepatic bile ducts [2]. While its incidence has markedly declined in the era of effective ART, dropping from approximately 20%-30% in the pre-ART era to less than 1%-2% among patients receiving modern therapy, it remains an important differential diagnosis in patients with AIDS who present with cholestatic liver enzyme abnormalities or biliary obstruction [3].

The pathogenesis of HIV-associated cholangiopathy is complex and multifactorial. Opportunistic infections are the most commonly implicated triggers, with *Cryptosporidium parvum* being the most frequently reported pathogen [4]. Other organisms such as cytomegalovirus (CMV), Microsporidia, *Mycobacterium avium* complex (MAC), and *Isospora belli* have also been associated with this condition [5]. These pathogens can directly invade the biliary epithelium or elicit an inflammatory response, resulting in fibrosis, strictures, and papillary edema. However, in a subset of cases, no infectious agent is identified, raising the possibility of HIV-induced immune dysregulation, direct viral cytopathic effect, cytokine-mediated damage, or suboptimal ART adherence/incomplete immune reconstitution contributing to biliary tract inflammation [6].

Clinically, HAC may be asymptomatic or present with nonspecific symptoms such as right upper quadrant pain, jaundice, nausea, fever, or weight loss [1]. Laboratory findings often reveal a cholestatic pattern with elevated alkaline phosphatase and gamma-glutamyl transferase. Imaging modalities, particularly ultrasound and computed tomography (CT), typically reveal bile duct dilatation, and magnetic resonance cholangiopancreatography (MRCP) or endoscopic retrograde cholangiopancreatography (ERCP) is essential for the definitive diagnosis and classification of the cholangiopathy subtype [7].

ERCP also serves a therapeutic role, especially in relieving biliary obstruction via sphincterotomy or stent placement [3]. Nonetheless, the cornerstone of treatment remains the initiation or optimization of ART to restore immune function and prevent recurrence or progression [1]. The recognition of HAC is critical for timely intervention, particularly in settings where opportunistic infections remain prevalent or ART access is limited [8].

## Case presentation

A 58-year-old man with HIV (diagnosed in 2022), on bictegravir/emtricitabine/tenofovir, presented with four weeks of progressively worsening right upper quadrant and epigastric pain, jaundice, nausea, bilious vomiting, 10 kg weight loss, and melena. His medical history included hypertension, alcohol use, and insomnia.

Table 1 shows the laboratory findings on admission.

**Table 1:** Laboratory findings at hospital admission BUN, blood urea nitrogen; ALT, alanine aminotransferase; AST, aspartate aminotransferase; LDH, lactate dehydrogenase; pCO₂, partial pressure of carbon dioxide; pO₂, partial pressure of oxygen

Parameter	Value	Reference Range
Glucose	98 mg/dL	70-110 mg/dL
BUN	18.9 mg/dL	7-20 mg/dL
Creatinine	2.07 mg/dL	0.6-1.3 mg/dL
Total bilirubin	5.44 mg/dL	0.2-1.2 mg/dL
ALT	264 U/L	7-56 U/L
AST	134 U/L	5-40 U/L
LDH	269 U/L	140-280 U/L
pH (arterial)	7.37	7.35-7.45
pCO₂	43 mmHg	35-45 mmHg
pO₂	25 mmHg	75-100 mmHg
Amylase	679 U/L	23-85 U/L
Lipase	946 U/L	0-160 U/L

Laboratory findings on admission included elevated lipase and amylase, consistent with acute obstructive pancreatitis in the setting of a distal common bile duct (CBD) stricture (Table 1).

**Figure 1:**
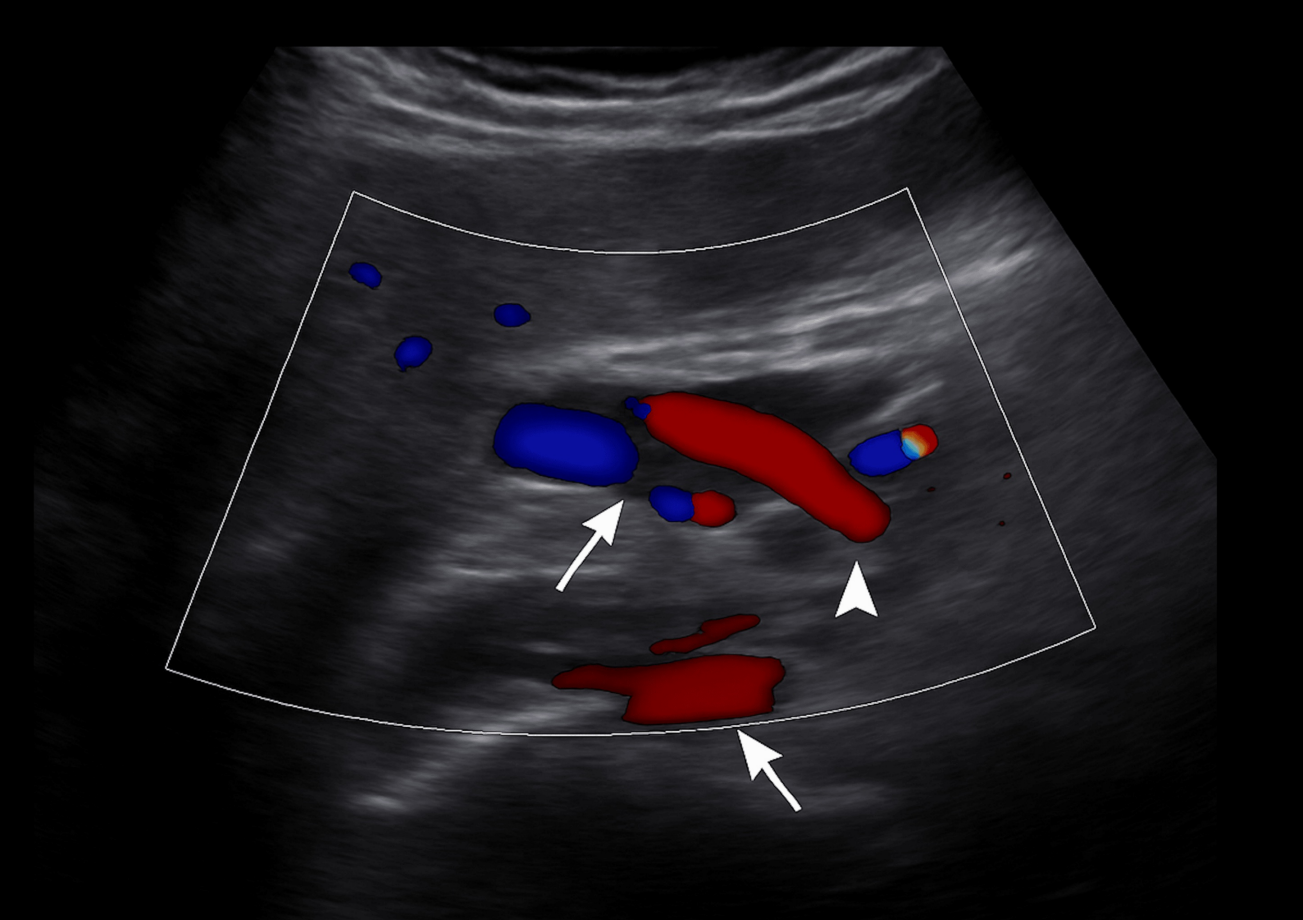
Doppler ultrasound image showing the intrapancreatic portion of the common bile duct (arrow) and the pancreas (arrowhead)

**Figure 2:**
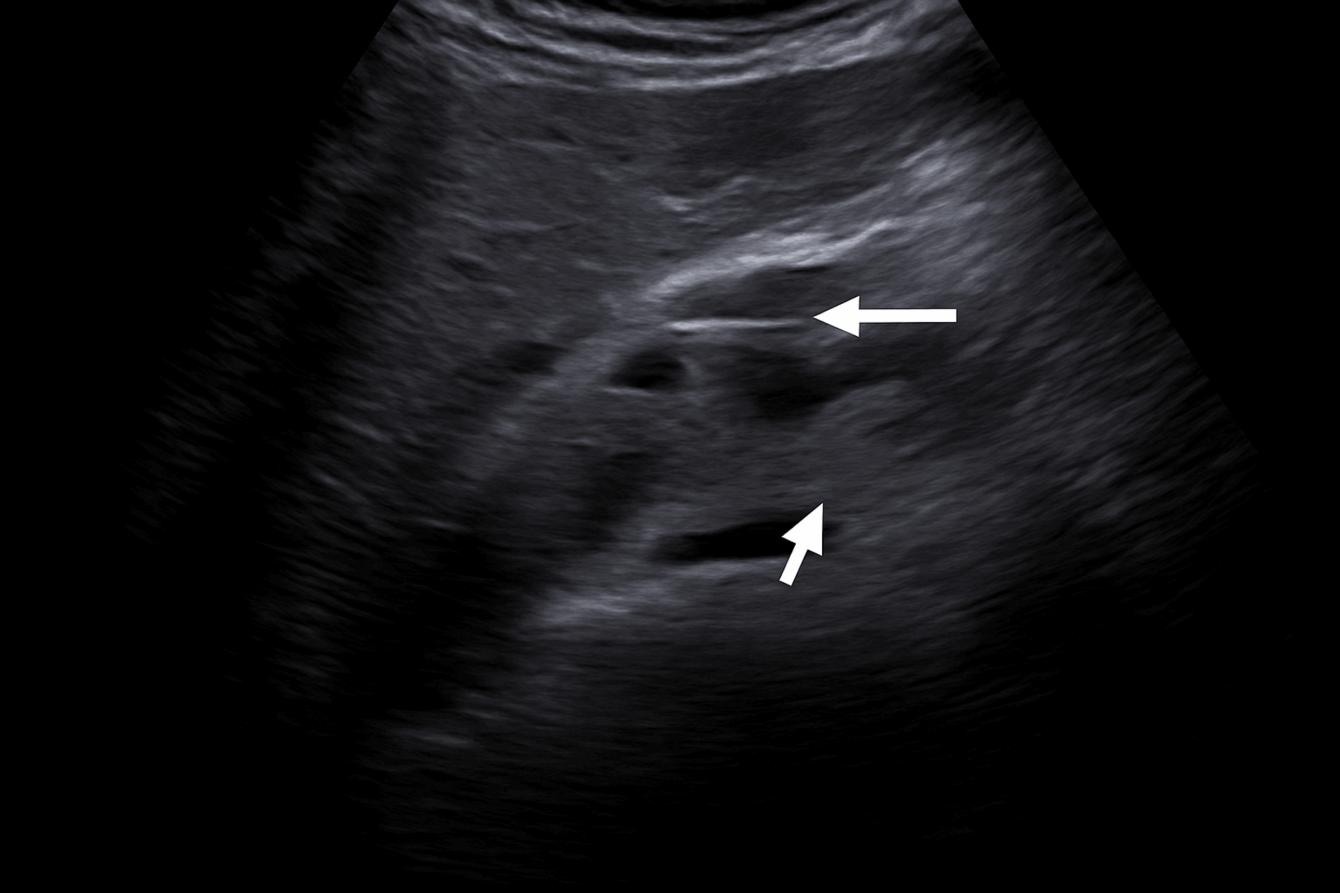
Doppler ultrasound image demonstrating the intrahepatic bile duct with a biliary catheter in situ (white arrow) The catheter appears as an echogenic linear structure within the common bile duct (CBD), confirming appropriate placement following endoscopic retrograde cholangiopancreatography (ERCP)

Ultrasound showed intra- and extrahepatic bile duct dilatation (CBD: 15.8 mm), the absence of gallbladder consistent with prior cholecystectomy, and a starry sky pattern. Doppler ultrasound demonstrated the intrapancreatic portion of the CBD and its vascular relations (Figure 1), followed by a confirmatory image with biliary catheter in situ (Figure 2).

**Figure 3:**
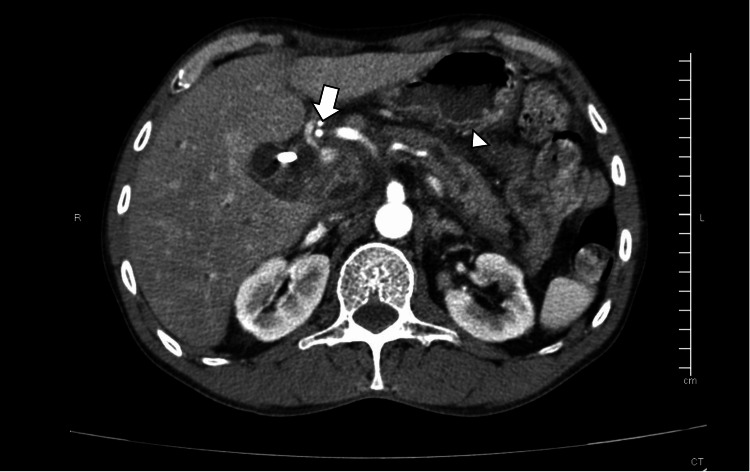
Axial contrast-enhanced CT scan showing a biliary stent (arrowhead) within the common bile duct and peripancreatic fat stranding with edema (arrow), consistent with acute pancreatitis CT: computed tomography

CT imaging showed biliary dilatation with an endoprosthesis and peripancreatic edema (Figure 3).

**Figure 4:**
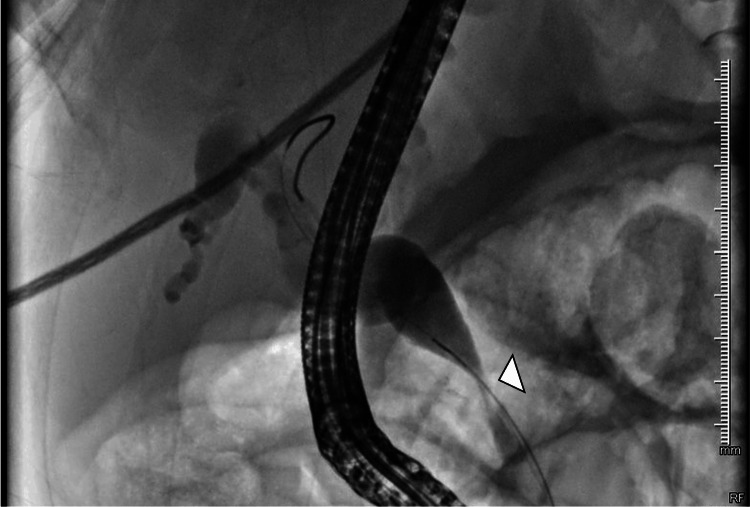
ERCP image showing distal common bile duct stricture (arrowhead) ERCP: endoscopic retrograde cholangiopancreatography

**Figure 5:**
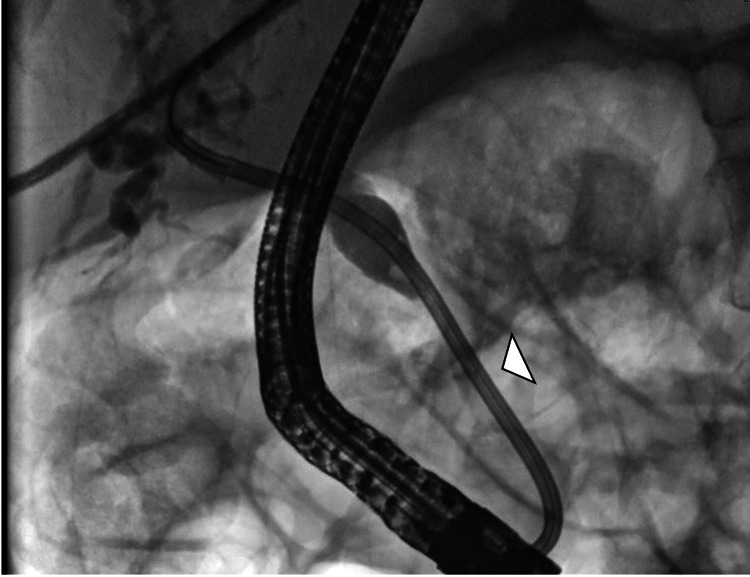
ERCP image showing biliary stent placement across the distal stricture (arrowhead) ERCP: endoscopic retrograde cholangiopancreatography

Given the clinical and imaging findings, a diagnostic and therapeutic ERCP was indicated, demonstrating a distal CBD stricture; a 10 Fr × 12 cm biliary stent was placed (Figures 4, 5).

Biliary brushing cytology was negative for malignancy (WHO category II), showing reactive atypia without dysplasia or malignant cells, and PCR testing for *Cryptosporidium*, CMV, and MAC from bile samples was also negative, supporting pathogen exclusion.

At discharge, follow-up laboratory tests showed significant improvement along with the normalization of electrolytes and a marked decrease in inflammatory markers, indicating the partial recovery of renal and hepatic function (Table 2). Alkaline phosphatase remained mildly elevated, which is expected to normalize gradually as biliary drainage continues. Follow-up imaging was scheduled to reassess ductal patency.

**Table 2:** Laboratory findings upon hospital discharge BUN, blood urea nitrogen; ALT, alanine aminotransferase; AST, aspartate aminotransferase; LDH, lactate dehydrogenase; MCV; mean corpuscular volume; MCH, mean corpuscular hemoglobin; M, male

Parameter	Value at Discharge	Reference Range
Glucose (mg/dL)	114	70-110
BUN (mg/dL)	15.3	7-20
Urea (mg/dL)	33.8	15-40
Creatinine (mg/dL)	1.46	0.6-1.3
Total bilirubin (mg/dL)	1.94	0.1-1.2
Direct bilirubin (mg/dL)	1.58	0.0-0.3
Indirect bilirubin (mg/dL)	0.36	0.2-0.9
Albumin (g/dL)	3.4	3.5-5.0
ALT (U/L)	80.5	7-56
AST (U/L)	55.2	5-40
Alkaline phosphatase (U/L)	861	44-147
LDH (U/L)	185	140-280
Calcium (mg/dL)	9.1	8.5-10.5
Phosphate (mg/dL)	3.0	2.5-4.5
Chloride (mmol/L)	91.1	98-107
Potassium (mmol/L)	3.7	3.5-5.0
Sodium (mmol/L)	132.2	135-145
Magnesium (mg/dL)	1.7	1.7-2.2
Hemoglobin (g/dL)	11.7	13.5-17.5 (M)
Hematocrit (%)	34.5	41-53 (M)
MCV (fL)	94.8	80-100
MCH (pg)	32.4	27-33
Platelets (×10³/µL)	711	150-450
WBC (×10³/µL)	9.38	4.0-11.0
Neutrophils (×10³/µL)	5.9	1.5-8.0
Lymphocytes (×10³/µL)	1.79	1.0-4.0
Amylase (U/L)	425	23-85
Lipase (U/L)	651	0-160

The patient showed clinical and laboratory improvement and was discharged under gastroenterology and infectious diseases follow-up.

## Discussion

HIV-associated cholangiopathy (HAC) is characterized by biliary inflammation and multifocal strictures, most often secondary to opportunistic infections in patients with advanced immunosuppression [1,3]. Common pathogens include *Cryptosporidium parvum*, cytomegalovirus (CMV), Microsporidia, *Isospora belli*, and *Mycobacterium avium* complex; however, in some cases, no infectious agent is identified, raising the possibility of direct HIV-related inflammation or immune-mediated injury [2,5]. Four anatomical subtypes have been described: papillary stenosis alone, intrahepatic sclerosing cholangitis-like lesions without papillary involvement, a combination of intrahepatic and extrahepatic involvement, and long-segment extrahepatic bile duct strictures with or without intrahepatic involvement [3]. These subtypes can often be visualized using imaging modalities such as magnetic resonance cholangiopancreatography (MRCP) or, more definitively, endoscopic retrograde cholangiopancreatography (ERCP). While MRCP is noninvasive and more widely available in some resource-limited settings, ERCP remains the gold standard as it allows both diagnosis and therapeutic intervention, which can be critical when access to multiple procedures is limited [6].

ERCP plays a dual role, offering both diagnostic clarity and therapeutic relief through interventions such as biliary sphincterotomy and stenting [4,6]. In our case, stent placement via ERCP provided symptomatic improvement in a patient who presented with a rare combination of acute cholangitis, obstructive pancreatitis, and acute kidney injury (AKI) in the setting of chronic kidney disease (CKD). This triad may serve as an early clinical indicator of HAC in immunosuppressed patients, prompting timely biliary evaluation and intervention.

Although the incidence of HAC has decreased in the era of highly active antiretroviral therapy (ART), it remains an important differential diagnosis in patients with advanced HIV and cholestatic liver enzyme patterns, particularly in regions with limited ART access or poor adherence [1,7]. The case also highlights that HAC may occur even in patients with undetectable CD4 counts, suggesting that viral load, immune reconstitution, and coinfections may influence disease expression.

Our case underscores the importance of maintaining a high index of suspicion for HAC in patients with advanced HIV who present with biliary obstruction, even in the absence of confirmed coinfections. While ART remains the cornerstone of management to restore immune function and prevent disease progression, endoscopic interventions provide effective symptomatic palliation and improve quality of life [1,4,6].

## Conclusions

HIV-associated cholangiopathy (HAC) should be considered in patients with advanced HIV infection who present with cholestatic liver enzyme patterns and clinical or radiological evidence of biliary obstruction, even when coinfections are not confirmed. This case also underscores that HAC may occur despite antiretroviral therapy, reinforcing the importance of adherence monitoring and immune reconstitution assessment in long-term management. While opportunistic pathogens, such as *Cryptosporidium parvum*, cytomegalovirus, and *Mycobacterium avium* complex, are frequent precipitants, there is growing evidence that direct HIV-induced inflammation and immune-mediated injury may also contribute to disease development. Recognizing this condition early is critical, as prompt evaluation can guide interventions that significantly impact patient outcomes. In this regard, initial imaging with ultrasound or CT scan serves as an accessible and effective tool for detecting biliary abnormalities, enabling timely referral for more definitive diagnostic procedures.

Endoscopic retrograde cholangiopancreatography (ERCP) plays a central role, offering both diagnostic precision and therapeutic benefit through interventions such as biliary stent placement, which can rapidly relieve obstruction and restore biliary drainage. Antiretroviral therapy (ART) remains the cornerstone of long-term management, aiming to reconstitute immune function and reduce the likelihood of recurrence or progression. This case highlights the importance of a coordinated multidisciplinary approach, engaging infectious disease, gastroenterology, radiology, and endoscopy specialists, to ensure comprehensive care. The integration of ART, targeted endoscopic therapy, and supportive measures not only addresses the immediate complication of obstruction but also improves the overall prognosis, underscoring the need for vigilance and collaboration in managing complex HIV-related hepatobiliary disease.
